# Why on earth did I buy that? A study of regretted appliance purchases

**DOI:** 10.1098/rsta.2016.0373

**Published:** 2017-05-01

**Authors:** T. Roberts, A. Hope, A. Skelton

**Affiliations:** 1 Department of Sociology, University of Surrey, Guildford GU2 7XH, UK; 2 Department of Engineering, University of Cambridge, Cambridge CB2 1PZ, UK

**Keywords:** regret, consumption, sustainability, well-being

## Abstract

If targets to reduce greenhouse gas emissions and thereby tackle climate change are to be achieved, it will be necessary to reduce both embodied energy costs (e.g. in terms of producing and manufacturing the products and services that society consumes) and operational energy costs. Reducing the number of purchases that people regret could be a first step in changing the overall dynamic of consumption patterns. This research looks at some potentially adverse effects of consumption on well-being (e.g. negative emotions), applying social practice theory to give insights into why people make purchases that they feel negatively about. This paper draws from: (i) findings of a national survey of over 2000 respondents which found that 53% of adults had reported regretting purchasing an electrical device at some point, and that 23% regretted making such a purchase within the past year; and (ii) a series of walking interviews around people's homes that provide detailed insights into the nature and extent of regretted purchases of electrical goods (e.g. resentment at built-in obsolescence, frustration at the pace of technological change). By combining the qualitative and quantitative data, we develop a typology of regretted consumption and explore the underlying factors that lead to such purchases. The paper concludes with a discussion of the policy implications of this research.

This article is part of the themed issue ‘Material demand reduction’.

## 1. Introduction

Domestic energy consumption accounts for the second largest proportion of final energy consumption (29%) in the UK after transportation (40%), and in 2015 showed the greatest increase in consumption (exceeding that of the transport, industry and service sectors) in both absolute and percentage terms [1]. In the UK, the domestic sector has experienced a year-on-year rise in electricity use of around 1% since 1970 [2]. This increase can at least in part be attributed to the growth in the number of appliances that people own and use. The number of appliances in homes has tripled since 1970 and has continued to increase by around 3% per year [2]. These changes have predominantly occurred due to the growth in the ownership of two groups of appliances, namely white goods (e.g. washing machines, dishwashers, fridges and freezers) and ICT equipment (e.g. computers, tablets, smart phones, televisions and set-top boxes) [3]. While there is some evidence to suggest that white goods are becoming more efficient and thereby using less electricity [4], the energy demand associated with ICT is rapidly increasing with growing public demand for these goods and services. Data centres, consumer devices and voice and data networks already account for 2% of global CO_2_ emissions, and, of these, data centres are expected to grow most rapidly [5]. As Andrae & Edler [6] note, by 2030 ICT could use as much as 51% of global electricity and contribute up to 23% of globally released greenhouse gases (GHGs).

The number of electrical appliances found within domestic settings is increasing, with a consequent increase in embodied emissions (i.e. from manufacture and production). The average UK household spends approximately £800 per year on electrical and electronic goods [7]. By weight, this amounts to around 1.4 million tonnes per year across the UK [7]. Research by WRAP and the Product Sustainability Forum has identified five product groups that have the greatest negative impacts (e.g. in terms of GHG emissions and the amount of resources used). These are televisions, washing machines, laptops, refrigerators and freezers, and mobile phones [7,8]. In short, the number of appliances in homes is increasing, and the types of appliances commonly being bought are highly resource-intensive.

Furthermore, the actual working lifespans of many of these resource-intensive appliances is decreasing; that is, more appliances are operating for shorter periods of time. For example, around one-third of washing machines and fridges in the UK are replaced each year because they fail to meet the average customer's expectation for each product's lifetime [7]. Similarly, consumers believe that their laptops are reaching the end of their useful/desirable life at around 2 to 3 years of age [7]. One reason for this is the pace of technological change, which can render otherwise functional products defunct. Another example is the ‘old’ analogue television and radio equipment which is being replaced by new digital technology, with the result that even while the equipment still has the capacity to work, it can no longer receive a signal. Christensen & Røpke [9] argue that these changes have led to the construction of a new ‘normality’ in everyday life where: ‘… the expectations and conventions regarding a normal house's “necessary” infrastructure and the ordinary gear for a normal way of life are changing, and the changes are proceeding rapidly’*.* In short, these technological changes are associated with changing cultural practices and social expectations, which in turn drive further technological change.

This proliferation of consumer electronics and domestic appliances has fundamentally shifted the way that people go about performing many everyday practices, such as doing shopping, preparing food, doing laundry and even communicating. These socio-technical changes have been directly attributed to significant improvements in well-being (e.g. time- and labour-saving devices such as washing machines). The implication of this is that any attempt to directly reduce the prevalence and use of technical innovations in society could potentially risk well-being.

Furthermore, reducing these products and services would also be contrary to market forces, which play an important role in dictating the direction of government policy. The principle that the current level of demand for new goods is desirable is also applied to the majority of environmentally extended economic models that are used to formulate options for reducing GHG emissions [10]. For instance, rather than focusing on demand-side response (e.g. households reducing or shifting their energy use), emphasis has tended to be put on supply-side management (e.g. additional power stations coming online to meet peak demand). This was even the case in the IPCC's 5th Assessment Report, where, out of the 1000 emissions pathways considered, 87% of the scenarios consistent with limiting warming below 2°C required net negative emissions delivered by supply-side carbon sequestration technologies (i.e. technological rather than behavioural solutions) [11].

Nonetheless, in order to restrict temperature increases to below 2°C above pre-industrial levels as agreed at the COP21 conference in Paris 2015, rapid and significant reductions in emissions of the order of 10% per annum among wealthier, industrialized nations are required [12]. Given that the development and construction of low-carbon energy infrastructure is likely to take many years, this will be unachievable if we rely solely on supply-side solutions [12]. There is, therefore, an urgent need to explore a wider variety of options for reducing energy demand (both operational and embodied) within the constraints of the current economic framework, and without reducing well-being.

In their paper, ‘Questioning demand: a study of regretted purchases in Great Britain’, Skelton & Allwood [10] propose that at least part of the solution to managing demand might be to tackle high levels of consumption that people later regret. The authors argue that regretted purchases indicate that the current level of demand is not necessarily desirable and, moreover, that if post-purchase regret is found to be widespread, regretted purchases may be used as a prompt to encourage people to question their demand patterns more generally [10]. Their survey on regretted consumption (which was administered by the pollster Yougov and completed by over 2000 respondents) identified that the vast majority of adults in Great Britain have regretted purchases at some point (82%) and the majority (68%) have regretted at least one purchase in the last year. Furthermore, it is likely that respondents under-report regret in a survey in a bid to maintain a sense of consistency between their beliefs, attitudes and behaviours and to reduce uncomfortable feelings of dissonance [13]. People may be reluctant, for example, to admit to themselves or to others that they have made a poor judgement in their purchasing decision. In short, there is a strong possibility that levels of regretted purchasing are even higher than suggested by the survey.

This paper takes the findings of Skelton & Allwood [10] as a starting point in further exploring the concept of regretted consumption. This is achieved by examining a number of domestic social practices associated with the proliferation of energy-consuming appliances in homes. In particular, we focus on the commonly performed practices of doing laundry, using ICT equipment and watching television. We combine analysis of the survey data presented in Skelton & Allwood [10] with data collected from 56 (qualitative) walking interviews. In the interviews, participants gave the researcher a guided tour of their home and answered detailed questions about their purchase and use of energy-consuming appliances.

## 2. A practice theory perspective on regretted consumption

While there is ‘no unified approach’ to practice theory, there is a growing consensus around Shove and Pantzar's understanding of practices as comprising three different elements which are linked together in order for a practice such as laundry to be performed [14,15]. ‘Materials’ is the first of these elements and includes: objects (e.g. washing machines), infrastructures (e.g. gas, electricity and water supplies) and the human body itself (i.e. in terms of the skills and physical capacity required to do laundry). The second element is ‘competence’. This can be understood as practical knowledge or skills (i.e. knowing *how* to do laundry). The final element is ‘meaning’. For Shove and Pantzar this is a combination of what Reckwitz describes as mental activities, emotions and motivational knowledge. In other words ‘meaning’ encompasses the goals or aims of the person performing the practice (e.g. wanting to have clean clothes), and also social and cultural standards (or norms) in terms of what it means, in this case, for someone to ‘be clean’ or to have ‘clean’ clothes [16]. It is important to bear in mind that practices do not remain static but are constantly evolving and changing as their elements shift. The introduction of washing machines, for example, resulted in new ways of doing laundry (e.g. mangles becoming redundant and washing machines becoming commonplace) and changed standards of cleanliness (e.g. the frequency of washing clothes).

By performing practices such as ‘doing the laundry’ practitioners are associated with: inconspicuous consumption (e.g. embodied energy costs in manufacturing and the energy consumption of the appliances); conspicuous consumption (e.g. ownership of washing machines, tumble dryers, detergents); the act of making a purchase itself; and the use of services [17]. Instead of focusing on any one of these particular ‘moments’ in or aspects of the life cycle of products, practice theory takes a more holistic approach. It focuses on the social and cultural context of which products and services are a part. Practice theory can be seen as a ‘big picture’ approach, focusing its lens on what social and cultural factors are in fact creating demand. What distinguishes a practices approach is that it considers the use of energy and the act of purchasing goods simply as ‘moments’ in the complex on-going and evolving performances of everyday practices, such as maintaining personal hygiene or communicating [18].

In our analysis, we focus on how the changing nature of domestic social practices encourages consumption. For example, we find that people feel compelled to purchase new devices even when they would rather continue to use their existing ones. We explore the idea of regretted consumption and how it can be manifested through a range of negative emotions. In doing so, we move beyond the traditional understanding of regret which has primarily been focused on the individual and their choices (see e.g. [19,20]), and take a broader view considering the role of societal pressures and evolving social norms. In short, rather than using behavioural models and looking at individual decision-making, we place greater emphasis on the cultural context in which products are purchased and used. The paper concludes by exploring a number of possibilities for the reconfiguration of everyday practices in ways that may be less reliant on the constant acquisition of additional devices and the regular upgrading of existing ones.

### (a) Causes of consumption

Social scientists have a long history of studying consumption. Much of this work has focused on studying the ‘consumer’, the process of exchange and the role individuals play in this process. In essence, the basic story given is that of individuals negotiating their way through institutional contexts over which they have limited control [18]. While many studies have taken this approach, generating some interesting and useful insights into the process of acquiring goods and services (see e.g. [21–23]), we argue that consumption is rarely performed in isolation and so a broader perspective is required. In short, consumers are normally *doing something*, or at least putting in place the necessary equipment to allow them to do something in the future (e.g. watching TV, cooking, doing the laundry) [24]. Therefore, it makes sense for the starting point of study to be the use of these goods and services, paying less attention to the economic exchange itself and more to the social organization of activities through which items are incorporated, deployed and disposed of [18]. By taking this approach the logic of consumption is found not in the selection of items but in the practices within which they are used [18]. This fundamentally changes the focus of consumption research. It directs research attention to a wide range of social processes, relations and interactions, and focuses on the ways in which people accomplish the tasks and practices that make up their daily lives. This approach is far broader than specifically focusing on the point of exchange.

Practice theory offers an alternative to the traditional economic and behavioural approach to the study of consumption. It emphasizes the role of routine and recognizes (i) that much consumption is largely invisible (e.g. use of electricity) and (ii) that it is the unintended consequence of the performance of everyday practices (i.e. getting things *done*). For instance, electricity consumption is a by-product of practices, such as watching television and sending emails [25]. This point is expressed very clearly by Sarah Pink: ‘…while I might suggest sitting on a comfortable sofa to watch a film with my family, I would not suggest that we sit together and consume energy’ [25].

While it may be relatively easy to apply a practices approach to understanding consumption of ‘invisible’ resources (e.g. electricity), applying this approach to more overt purchases (e.g. buying large and/or expensive consumer electronics) requires further consideration. Let's take the purchasing of a washing machine as an example of how a practices approach can be helpful in understanding consumption. We can begin by considering the structural aspects of social practices related to cleanliness and the maintenance of domestic appliances in the home. Cultural expectations relating to cleanliness are not explicit. Nonetheless, people have a sense of what constitutes a ‘right’ way of doing laundry and of how they should present themselves to others (e.g. avoiding wearing clothes that might risk being perceived as dirty, smelly or simply not fresh) [26,27]. As the social cues related to acceptable levels of cleanliness are difficult to communicate and receive, people generally adopt a ‘precautionary approach’ and go beyond the minimums levels of cleanliness necessary [28]. Cultural expectations relating to what constitutes an acceptable level of cleanliness for clothes have evolved over time, and continue to do so as a direct result of socio-technical change (e.g. automatic washing machines). As the amount of time required to undertake laundry has decreased through the introduction of automatic washing machines, cultural expectations of what constitutes an acceptable level of cleanliness have increased, with people washing their clothes more frequently [28]. This process of socio-technical change is fundamental to understanding the constant evolution of everyday practices. This reconfiguration of laundry practices has also been a major driver for the acquisition by households of automatic washing machines. These are now deemed necessary if one is to conform to contemporary conventions related to living a ‘normal’ everyday life. Around 95% of households in England now, for instance, have a washing machine [29]. It can be seen from this example how a practices approach can help to illuminate the context in which an individual makes a purchasing decision, and how an individual's decision is shaped by factors that extend far beyond the self.

The same principles of investigation can be applied to the consumption of other consumer electronics. For instance, advancements in mobile phone technologies have co-evolved with cultural expectations about connectivity and accessibility, in turn driving demand for the latest technologies to enable people to conform to the ever-evolving cultural conventions. In short, it is often the subconscious desire to conform to cultural expectations that drives consumption rather than explicit individual choice.

### (b) Obsolescence of appliances

Reasons for the disposal and replacement of electrical products do not simply relate to the natural depreciation in value and functionality of products (i.e. end of ‘working-life); they also relate to their perceived desirability.

Analysis of the obsolescence of appliances can be conducted at a number of levels. Packard [30] describes three categories of obsolescence which are still relevant today when exploring drivers for consumption. First, there is *obsolescence of quality,* or a lack of quality components or materials, which determine the durability of the product. Second, there is *functional obsolescence*, which is induced by new innovations, new features and new interfaces. A good example of this is the switching off of the analogue television signal in the United Kingdom, which rendered many older (but otherwise functional) televisions obsolete. Third, there is the *obsolescence of desirability*, which is inspired by a desire for new devices, designs and lifestyles. This results in products becoming perceived as old-fashioned and outdated. For example, developments in the digital storage of music have led to large hi-fi systems capable of playing music in multiple formats (tape, compact disc, record, etc.) being replaced with small sets of speakers to which portable mp3 players can be connected. By looking at the way in which products become obsolete from these three perspectives, it is possible get an indication of how the evolution of everyday practices through socio-technical change results in the consumption of new products.

### (c) Regretted consumption

If consumption is considered as the process of acquiring the necessary equipment to perform social practices, the concept of regretted consumption may on the surface seem to be focused on individual decision-making (i.e. the individual regretting *their* choice). However, tastes and preferences are socially formed and preferences are determined not just by the available economic resources but also by cultural competence (cultural capital), social connections (social capital) and social reputation (symbolic capital) [13,25].

By applying the *concept of habitus* (understood here as the physical embodiment of skills, habits and dispositions gained through the course of a person's life experience), Bourdieu [31] challenged traditional accounts of the individualization of lifestyles (e.g. the idea of individual lifestyle *choices*) by emphasizing the role of embodied practical reasoning (i.e. culturally determined ways of doing things) and entrenched dispositions (i.e. culturally informed/determined preferences). This has important implications for the study of consumption (i.e. *why* people make purchases) and has been fundamental in the development of practice perspectives on consumption. Later work adopting the practice perspective, for example, rejects the idea of the sovereign consumer and instead places emphasis on doing rather than thinking, on the material rather than the symbolic, and on embodied practical competence (e.g. from participating in something), and the flow and sequence of routines, rather than discrete individual actions [31]. Nevertheless, to date, the majority of analyses have been conducted from economic and psychological perspectives [32] where emphasis has been placed on individual choice in purchasing decisions. It is through this kind of framework that ideas about the impact of regretted consumption have been developed. Consequently current understandings of regret are focused on an emotional state related to (misjudged) individual choices. Here, however, we adopt a slightly broader understanding of the term ‘regret’ which encompasses both the cultural and social dimensions of practices in order to provide a new and potentially useful angle on the analysis of consumption practices.

Before we move on to take a broader view of regret it is first necessary to explore traditional understandings of the concept. Zeelenberg & Pieters' [33] definition provides a useful starting point. They describe regret as: ‘the emotion that we experience when realizing or imagining that our current situation would have been better, if only we had decided differently. It is a backward looking emotion signalling an unfavorable evaluation of a decision. It is an unpleasant feeling, coupled with a clear sense of self blame concerning its causes and strong wishes to undo the current situation.’ Skelton & Allwood [10] go on to apply this definition to regretted consumption, arguing that, within this definition, regretted purchases could take multiple forms (e.g. ‘I wish I hadn't bought it’, ‘I wish I hadn't bought this one, I should have chosen a different one’, ‘I frequently regret one particular purchase’ or ‘I wish I had bought more’).

However, if consumption is an enabling element which allows us to perform a particular practice, we need to ask whether regret can also be attributed to the *performance of a practice,* rather than just the consumption aspect of it. In particular, we need to look at the emotions people express about the pressure to conform to a constantly evolving set of social and cultural conventions, which regularly require the acquisition of a changing set of ‘equipment’. A good example is the change in personal communications technology over the past 5 years. According to OFCOM [34], in the UK 71% of adults now own and regularly use a smart phone compared with 26% in 2010. This has led to a fundamental reconfiguration of one of the most basic everyday human practices, namely, communication. According to OFCOM, even relatively new forms of communication such as e-mailing and texting are now in sharp decline, instead being replaced by instant messaging and social media. Consequently, it is becoming increasingly difficult to partake in basic everyday activities without access to smart phone technology. Such fast-paced socio-technological change may leave some people feeling pressured to purchase particular products in order to enable them to continue playing a full and active role in everyday life. Regret, therefore, may come in a number of forms: regret at dedicating an increasing amount of time to the practice of electronic communication; regret at having caved in to social pressure to purchase a smart phone; and regret at not having waited longer to purchase a smart phone and thereby having missed out on having the very latest model with its new functionalities.

## 3. Material and methods

As highlighted in the literature, any attempt to restrict temperature increases to below 2°C above pre-industrial levels, as agreed at the COP21 conference in Paris 2015, will require a significant shift in contemporary consumption practices. To date, scholars, particularly those working from a practice theory perspective, have made significant progress in understanding what drives consumption, but there are still significant gaps in our understanding. In the results presented below, derived from two separate but related studies, we attempt to further our understanding of one of those knowledge gaps, regretted consumption.

In this section, the data collection and analysis techniques are briefly described. More details of the methods used to generate the quantitative data can be found in Skelton & Allwood [10] and a more detailed account of the qualitative data collection, namely walking interviews, will be published in a forthcoming paper.

### (a) Quantitative data collection

A series of questions developed by researchers at Cambridge University on consumption practices were included in the omnibus survey run by the pollster Yougov. The omnibus survey is run daily and consists of a medley of questions from different research projects that are put to a pre-selected panel of over 2000 respondents who are given a small financial incentive to complete the survey. This particular omnibus survey was run on 19 March 2015 and took approximately 15 minutes to complete online. Respondents were paid 75 pence for their participation. The survey included a wide range of questions about regretted consumption related to a range of different product categories. There were also a range of questions from other research projects focusing on issues including: private medical insurance, UK oil and gas, and the Discovery Channel. Responses to this particular survey (presented here) were obtained from 2036 people. A full report providing further details of this study design and results is set out in a separate paper; see Skelton & Allwood [10].

The data presented in this paper are from a small subset of questions related to the purchase of electronic items and is focused on the questions outlined in table 1.

**Table 1.** Survey questions.

question	sub-question	response
Thinking of when you have bought the following products in the past. How often, if ever, did you later regret your purchase? Please choose the option that best applies. This question was preceded by the statement: For the following question by ‘regret’, we mean you wished that you hadn't bought something in the first place, for any reason.	— Electronic devices (e.g. mobile phones, cameras, tablets, e-readers, games consoles and TVs) — Kitchen gadgets (e.g. bread makers, pasta makers and mixers) — ‘White’ goods (e.g. fridges, washing machines)	— More than a couple of times in the last year — A couple of times in the last year — Just once in the last year — In the past but not in the last year — I've bought this product, but never regretted it — Don't know/can't recall — Not applicable. I've never bought this type of product.
You said that you have regretted buying [product group] in the past. Why did you regret making these purchases? Please select all reasons that apply.		— On reflection, I couldn't really afford it — I was enticed by an offer or an advert and didn't really need it — It wasn't right for me after all or wasn't as good as I expected it to be — I didn't use it as much as I expected to — On reflection the product didn't fit with my wider health, environmental or social concerns — I later found something out that made me regret my purchase (e.g. I saw it on offer or saw another superior product) — Other, please specify… — Don't know/can't recall.

### (b) Qualitative data

#### (i) Collection and sample

The qualitative data presented here come from two separate studies looking into social practices around domestic energy demand. A total of 36 interviews were undertaken for the Engineering and Physical Sciences Research Council (EPSRC) funded Whole Systems Energy Modelling Consortium project. These data were collected between January and April 2013, and November 2015 and April 2016. Participants were based in and around the city of Guildford, Surrey, in the South East of the United Kingdom. Participants were recruited through advertisements placed on a number of online community forums. In return for participation participants were compensated between £20 and £100 (depending on which stage of the research they were involved in). A further 18 participants were recruited as part of the British Academy funded ‘Out of Sight, Out of Mind: The problem of invisibility for environmental policy’ project. These data were collected between October 2015 and April 2016. Participants were based in villages along the north Norfolk coast. Participants were recruited through advertisements placed on an online community noticeboard and flyers handed out at community events and meetings. Participants were compensated £20 for their time.

Because the two research projects were interested in exploring participants’ perceptions of the impact of internet access on everyday domestic practices, only those over 35 years of age were invited to participate in the studies. This was to ensure that all participants had at least some experience of domestic life as an adult before the widespread roll-out of fast broadband internet connections in domestic homes. While our sample is not representative of the UK population, it nonetheless includes a variety of household types and tenures (private ownership, social and private rentals), ranging from small rented studio flats, to large owner-occupied detached properties. The majority of the interviews were carried out with a single occupant of each of the properties. On a few occasions, however, participants requested to be accompanied by their partner. Furthermore, because the interviews involved a tour of each household, the voices of other household occupants are also sometimes present (e.g. as household members discuss together the question posed by the interviewer).

#### (ii) Procedure and interview schedule

The interviews were semi-structured, that is, the researcher had a predefined set of questions to discuss with the participant. While the schedule was there to guide the discussion, the researcher was able to adapt the interview schedule as required (e.g. asking additional questions, exploring related points of interest which emerged during the interview). Data were collected until it was felt that no new themes were emerging from the interviews.

The interviewer began by asking a series of questions about the participant's energy consumption practices. This was done while seated in a communal area of their home (normally the kitchen or living room). Following this, there was a walking interview around the home. During this phase of the interview participants were asked to lead the researcher through their typical daily routine, highlighting where and how they performed specific energy-intensive practices, such as: communication; maintaining thermal comfort; visual entertainment; laundry; and food preparation. During the tour the researcher asked a range of questions (e.g. relating to energy use, perceptions of renewable energy technologies) but only a subset of relevant questions were selected for analysis for this research. These are presented in table 2.

**Table 2.** Interview questions.

Questions asked about energy-consuming devices during the walking tour
1. When did you purchase [device]?
2. Why did you purchase them [device]?
3. How do you use [device]?
4. Is this how you thought you would use them [device]?
5. Do they live up to your expectations [device]?
6. Would you buy them again? If not why? Would you look for something different [device]?
7. How long do you expect them to last for [device]?
8. Do you regret buying the device?

It is worth noting that the tour was predominantly led by the respondent, and discussions about particular devices were normally instigated by the respondent. A key advantage of this approach is that it enabled the researcher to ask probing questions about devices which were seen during the tour but which were not highlighted by the respondent. In terms of asking questions about regretted purchases, this approach was particularly useful, as participants tended not to highlight unused or obsolete purchases without being prompted. Another notable advantage with the walking interview is that it allowed for the participants to talk about often mundane everyday practices and the ‘equipment’ used to perform them in the correct context. The household context not only provided visual aids but also encouraged participants to provide more in-depth and detailed explanations.

#### (iii) Analysing the data

The interviews were digitally recorded and transcribed verbatim by a professional transcription company. Following transcription, the main author conducted a thematic analysis of the interviews. Some themes were established *a priori* because the interviews were semi-structured, meaning that there was a set of predefined discussion topics. The process of thematic analysis was assisted by qualitative software, in this case NVivo 10.

## 4. Results

This section first reports findings from the survey and then moves on to the interview findings. These two datasets focus on the equipment necessary to undertake commonly performed (and often interconnected) everyday practices (e.g. laundry, electronic communication and visual entertainment). The interviews give insights into the process of purchasing the necessary equipment to perform these (and other related) practices, and importantly into why people sometimes express post-purchase regret after the acquisition has taken place.

The quantitative survey data are explored (i) to gain insights into the extent of post-purchase regret of electronic appliances and the reasons people stated for regretting a purchase and (ii) to develop a more in-depth understanding of the different ways people express regret.

### (a) Survey results

**Figure 1.**
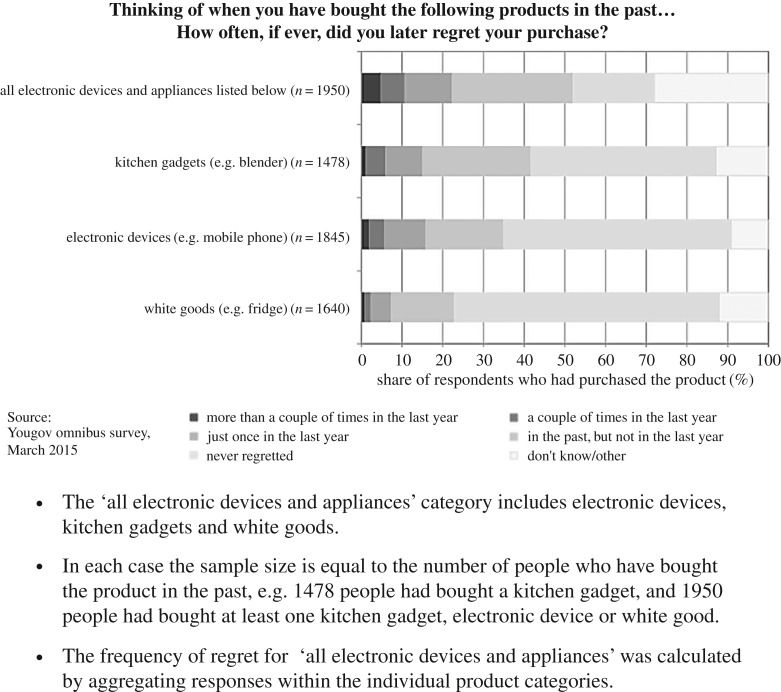
Frequency of regret by type of electronic device.

The survey showed that 82% of adults reported a regretted purchase in at least one of the product categories (e.g. electrical devices, clothing and footwear, takeaways and vehicles) in the past, and 67% regretted a purchase within the past year. According to Skelton & Allwood [10], it was estimated that these regretted purchases cost households a total of £430 per year. Specifically, in terms of electrical devices, 53% of adults had regretted a purchase in the past and 23% had regretted a purchase in the past year. Figure 1 shows that, when broken down further into product categories, kitchen gadgets were the most frequently regretted purchase. In total it was estimated that households spent £61 per year on these particular regretted purchases.

**Figure 2.**
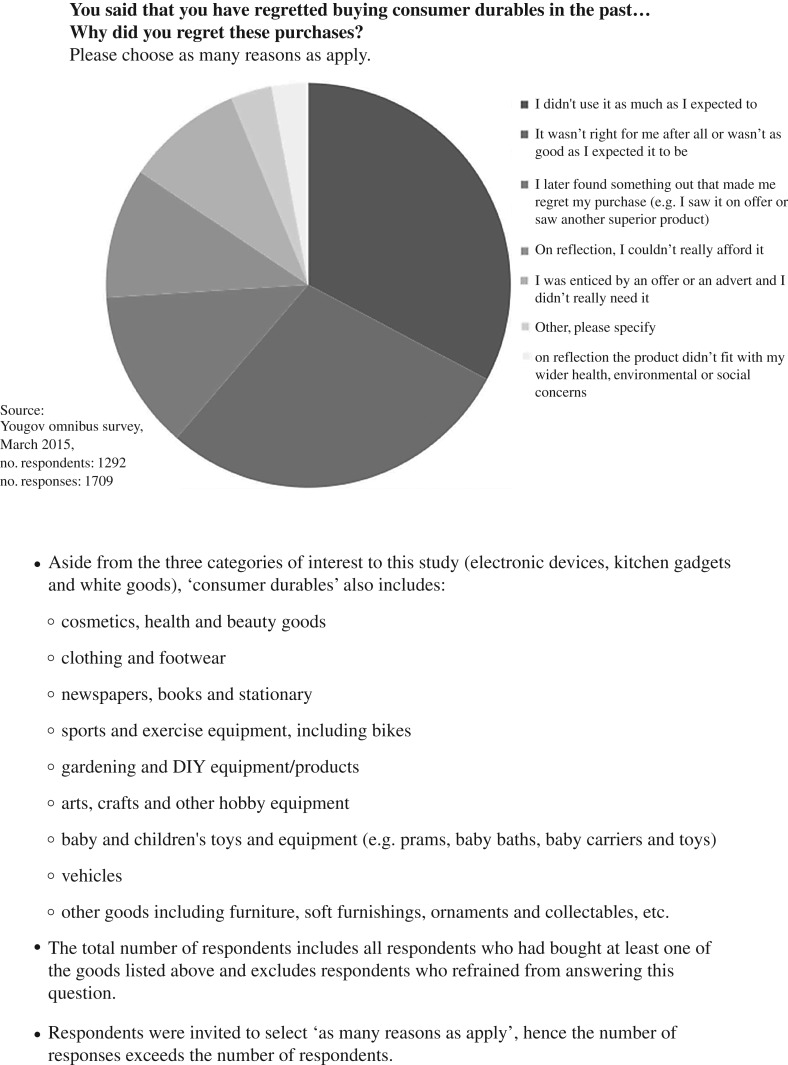
Reasons for regret.

Respondents who had indicated that they had regretted purchases were asked why this was the case. They selected as many reasons as applied to them from a set list. Figure 2 shows that in the case of consumer durables (which includes a wide range of household products in addition to the electrical devices listed in figure 1), the primary reason for regret was that the respondents did not use the device as much as they expected (33%), closely followed by a concern that the product did not perform well or as expected (28%).

Relatively few respondents (approx. 3%) selected the ‘other’ reasons option for regret and most of the comments provided fell within the survey categories already provided (figure 2). However, these comments do give us further insights into the areas that were of most concern to the respondents. In terms of electrical devices, the quality and functionality of the product appears to have been the greatest concern. Examples of the reasons given by participants for regretted purchases of electric devices include the following: ‘It broke’; ‘Faulty goods’; ‘Disappointed with performance and quality of fridge and washing machine’; ‘ A number of design flaws that became apparent after purchase’; ‘It was lower quality than I thought’; ‘Noisy washing machine’; ‘Didn't function properly and became unsafe for my children’*.*

### (b) Walking interview results

Findings from the qualitative data collected during walking interviews corresponds with the survey findings and also provides a more in-depth insight into the way in which people think about the purchases they have made.

#### (i) Regrets, unhappiness and justifications

When participants were directly asked whether they regretted purchasing a specific electrical device, relatively few openly admitted that this was the case. It was clear that the vast majority of respondents felt that most of the devices they had in their homes were useful and necessary for performing everyday practices (the only exception being some kitchen appliances). However, this did not mean that participants did not express negative emotions about at least some of the purchases that they had made. While participants initially stated that they did not regret purchasing an item, they often went on to: (i) justify the purchase (a defensive response) and (ii) list a range of faults with the device. For example:

**Valerie, 55:** ‘[We] went out to buy a very basic large TV but got distracted. I'll be honest with you, we got distracted by [a] Smart TV thinking it was a good idea but we're still learning how to use it.’

**Interviewer:** ‘Do you have any regrets about buying this particular TV, or…?’

**Valerie, 55:** ‘No. The only regret was the cost because it was very expensive and they've now come down particularly in price and that was the one thing.’

Here the participant initially answers in the negative, but immediately goes on to express regret about the cost of the item; her regret being that she invested too soon in the appliance and could have saved money by waiting longer.

In addition, participants often justified the purchase of infrequently used and neglected appliances on the basis that the item *might* one day be useful (i.e. based on future intentions or potential utility):

**Interviewer:** ‘You said you don't use it [CD player] much but do you regret buying it?’

**Dawn, 56:** ‘Oh, not at all, no. It's nice that it's there and [if] I am in here for any reason, when my children come back and we have dinner we could have music on, so it's nice, yeah.’

Justifications also related to personal identity, for example: ‘I do buy stuff that I don't use very often. Yes, I'm a bit of a kitchen gadget freak’ (Jon, 61). Here purchases can be seen to have symbolic meaning, signalling the participant's interest in and engagement with technology. References were also made to the difficulty or impracticality of avoiding making a purchase, or to a lack of perceived control, for example: ‘Our fridge actually broke so we were forced to go and buy a new one’. These kinds of responses illustrate participants’ ambivalence towards the purchase of household appliances and reveal feelings of tension (i.e. not wanting to have negative feelings about a purchase or to regret it). In the latter quotation (from Jon, 61) the negative emotion is not at having bought a fridge *per se*, but rather at the necessity of *havin*g to spend money on replacing a broken item. In short, Jon *resents* having to make the investment.

#### (ii) Obsolescence of devices

Negative emotions were associated with ‘built-in’ or ‘planned’ product obsolescence. That is, participants were unhappy that products were designed to wear out or become outmoded after limited use. This was expressed in a number of ways. First (and especially with reference to white goods) there was a general sense, particularly amongst older respondents, that ‘modern’ appliances did not last as long as ones bought some time ago. In short, it was felt that these types of modern appliance would only be usable for a short period of time before having to be replaced:

**Pamela, 60:** ‘Well our washing machine… was replaced earlier this year or the end of last year and we reckon that was about 25 years old…. I don't reckon the new one will last more than 7 years, or something like that…. I was convinced that it [the old one] was on its last legs but it was better at getting stuff clean than the new one.’

When asked why she thought new products didn't last as long she stated that:

**Pamela, 60:** ‘the materials they used just aren't as good.’

Another respondent was even more explicit in his answer, arguing that contemporary devices tend to be built to fail, unlike older ones, which were built to last:

**Steve, 65:** ‘…they do have a little bit more built-in obsolescence, I think, than they used to.’

Second, negative emotion was related to concerns about the lifespan of the devices. Many of the respondents reported that they had recently replaced electronic devices because it was cheaper and/or easier to replace them rather than repair them. The two primary reasons stated for this were challenges in accessing spare parts, and the ‘sealed’ nature of many contemporary devices, meaning that access to the faulty part was difficult.

**Anne 60:** ‘We're an incredibly throw-away society. I think instead of repairing or reusing things, we are much more inclined to throw things away and buy something new; it's much easier, as I've discovered (as I had a television that stopped working), I couldn't get anyone to repair it, so it became easier to buy a new one.’

**Mark 57:** ‘They're [i.e., washing machines] very, very simple things to fix and it does annoy me that people just: ‘Oh it's not working, throw it away, I'll buy another one.’ Nobody ever bothers to look – ‘Oh it's the pump’. Well I can buy a pump for fifteen quid from the spares man and I can fit it and […], you know, a new washing machine. But no: ‘I'll go and spend £250 on a new washing machine.’ It doesn't make a lot of sense to me.’

This sense of frustration about the lifespan and disposability of devices was expressed in two ways. First, participants felt very negatively about the fact that they had had to purchase a new appliance earlier than they felt should have been necessary, and second, they were unhappy that they were replacing older, more reliable, appliances with newer, less reliable ones. To summarize, participants expressed a range of complex negative emotions (e.g. disappointment, resentment, frustration and regret) at the performance and durability of goods and the fact that they were having to invest money in replacing them so quickly.

#### (iii) Technological and social change

In addition to concerns about the quality of the products, a number of participants expressed negative emotions at having to purchase new products due to technological change. This was especially the case when participants felt the purchase was in some way forced upon them. This feeling was particularly prevalent when it came to ICT equipment. While many of the respondents reported still owning computers that were over 5 years old, there was a general consensus that these machines struggled to undertake many contemporary computing tasks due to their lack of processing power. ICT equipment was considered technically obsolete after about 3 years:

**Steve 36:** ‘I would probably, I would hope it would last at least three years. If it lasted five I'd be surprised. So, I would probably say three years and it's not so much in terms of it breaking or, kind of, wearing out, but in three years' time the software will require so much more processing power that a laptop of three years old won't really cut it any more.’

Rather than immediately replacing ‘outdated’ devices, many of the participants reported that there was overlap in the use of different devices. Old devices (e.g. desktop computer) continued to be used for some time alongside new devices (e.g. smart phones or laptops) which were more ‘up to date’ and able to meet new demands. The data suggest that, as practices change, materials that are rendered less useful are gradually phased out and, therefore, overlap with the development and adoption of new or revised practices (e.g. using a phone to check email). This process of acquiring new devices without disposing of old ones has led to a proliferation of devices within homes:

**Emma 37:** ‘We don't use them [laptops] that much to be honest because we've got phones, we can get on the internet on them, so you know, we don't use them half as much as we would have done a few years ago. But there are things you can't do on a phone, you need to download something or it's on a disc or things like that, so we tend to use them more to print something off, you know, but we don't really use them that much.’

The respondents also recognized that the purchase of new devices was not always driven by old devices breaking, becoming functionally obsolete or the introduction of new technology. Social pressure and a desire to conform to the latest trends were also a significant factor. As Carol notes, while talking about why she has replaced all her old ‘big’ televisions with flat-screen ones:

**Carol 47:** ‘These days people don't tend to replace things because they don't last, they tend to replace them because they want more technology.’

With the proliferation of devices in the home, many of the participants expressed negative emotions about new purchases and the functional demise of old ones. While the respondents generally embraced new technology and wanted to reap the benefits, the constant pressure to expand the number of devices they owned and keep up with the latest technology was often a source of frustration. Participants resented and generally felt negatively about having to make purchases which would not have been deemed necessary in the recent past (e.g. smart phone). Again, our participants didn't regret the purchases, as such, but rather felt frustration and resentment at the fact that they had become necessary to perform practices (e.g. needing a smart phone to communicate).

#### (iv) Other reasons for unhappiness with consumption

So far this research has identified a range of negative emotions through which the respondents expressed regret about their purchases. These related to obsolescence, under-utilization of products (e.g. not getting one's money's worth), investing too soon (e.g. before prices came down or the technology further developed) and social and technical pressures to keep updating goods (e.g. the need for iPhones/smart phones to keep in touch with people). However, a range of other reasons for regretting purchases were also identified by the interviews and survey. ‘Other’ reasons for regret were: buying products without first seeing them (e.g. via the phone or internet); not buying better quality products; buying products that turned out to be difficult to use; and the giving or receiving of gifts that were not appreciated (e.g. were perceived as useless). Table 3 provides a detailed overview of findings and a typology of the forms of negative emotion identified in this research.

**Table 3.** Typology of the negative emotions through which regretted consumption was expressed.

regret due to:		examples from the survey and walking interviews	solution
lack of information at purchase	on prices	(1) ‘Better price elsewhere’; (1) ‘Found it cheaper elsewhere’; (1) ‘Paid over the odds’	price comparison websites; ‘find cheaper elsewhere’ refunds; returns; reuse
	on chosen product	(2) ‘Colour not as shown in advert’	regulation on advertising; returns; reuse
	on alternative product options	(2) ‘Yes. I suppose looking at the cooker, this side is an induction hob and this side is just a ceramic hob; the reason for that was I couldn't get enough information on an induction hob […] I am now sorry I didn't go for a total induction because it's so fast and so effective’; (2) ‘I bought this on the phone […] I rung them up and asked them however they can give something an AA rating and take three hours to do the wash, but apparently that's on the spin and […] I thought that would be efficient but one could say one was misled, and the next thing one should not have bought one on the phone without seeing the bloody thing’	product labelling; try before you buy; returns; reuse
the performance of the product	immediate problems post-purchase	(1) ‘Noisy washing machine’; (1) ‘Faulty goods’; (1)’ A number of design flaws that became apparent after purchase’; (2) ‘It's [electronic onion chopper] actually more trouble than it's worth, it takes longer to prepare the onions to go in it than it does to chop them by hand’; (2) ‘Well functionality, ease of use; modern things are so complicated now, I have to get the instruction book out every time I want to set the flippin’ timer practically’	product labelling; try before you buy; returns; reuse
	degradation over time	(1) ‘It broke’; (1) ‘It became unsafe for my children’; (2) ‘The latest washing machine is on its way out, my previous one lasted twice as long’	extended warranty; product repair services; returns; reuse
	dependence on compatible products	(2) ‘[W]e had it put in [vehicle charging point], we were all intent on buying an electric car, […] did the test drive and everything, all ready to sign the papers and then realised that the batteries … that they only last so long and you need to replace them after so many years and it was so much money. […] So all our plans for buying electric cars went out of the window but we had everything ready for an electric car. So maybe in the future…’	
	poor auxiliary services	(1) ‘Delivery time kept going back’; (1) ‘I was sent the wrong order twice, one company put it right the other company promised a refund but after the 3 months they knew it was too late for me to do anything, I never got my refund. The furniture they sent me was £100 less than the one I bought from them!’; (1) ‘Not user-friendly ie instructions are on-line or otherwise too complicated to access/activate’	improved services; better access to instructions; customer services training
poor decision-making	misjudged need	(1) ‘I didn't need it’; (2) ‘I thought it would be useful but it turns out it's not’	try before you buy; returns; reuse
	changing preferences over time	(1) ‘I got bored with it’; (2) ‘Well I don't particularly regret … but I've got an ice-cream maker up there which my partner keeps saying, ‘Why don't you make some more ice-cream?’	reuse
	price/quality trade-off	(2) ‘We bought sort of at the bottom end so everything, you know, works and everything but like the oven and the hob and the washing machine, they're all kind of fairly cheap ones which drive Judy round the bend now because she wants a decent … I think the next thing to buy is a decent oven.’	awards for high performance products; returns; reuse
	under-valued alternatives	(1) ‘Wished I still had the money’	returns; reuse
	social pressures on purchases	(1) ‘Went with my husband's choice, not mine’; (1) ‘Advised by sales staff/friends/family that it was a good choice… this was not so’	comparison websites, information campaigns
	unappreciated gifts	(1) ‘The person I bought it for didn't like it’; (2) ‘They've all really been presents, either wedding presents or presents, and I think they might not be that worth having because use them like once a year’	gift receipts; returns; reuse

**Key.** (1) Example from open response question in quantitative survey, (2) quotation from walking interviews.

## 5. Discussion

The household survey indicated that the majority (53%) of adults in Great Britain have regretted the purchase of electrical devices at some point in the past, and 23% had regretted a purchase in the past year, costing households an estimated £61 per year. It was expected that these reported levels of regret might be an underestimation because people may be reluctant to admit to making a poor purchasing choice. The walking interviews were intended, therefore, to give broader insights into regret. What they found, however, was that regret was expressed through a complex range of negative emotions associated with consumerism, and more specifically the purchase of electronic devices. Combined with the analysis provided by Skelton and Allwood [10] these findings suggest that: (i) while the acquisition of material goods can promote well-being, these benefits risk potentially being undermined by regret expressed through a whole range of negative emotions (e.g. resentment, disappointment, frustration); and (ii) policy interventions to reduce levels of regretted consumption (or general unhappiness at consumption) in this area could potentially contribute to reducing energy demand (embodied and operational) without necessarily negatively impacting welfare.

In the remainder of this article, we will further elaborate on these findings. First, we attempt to provide a new and more comprehensive understanding of regret to apply to consumption, which emphasizes the social (as opposed to individual) dimension. Second, we consider some preliminary policy implications of this study, and finally we offer some suggestions for future work in this area.

### (a) Understanding unhappiness with consumerism

Gaining a better understanding of the negative emotions associated with the purchase of household electronic appliances and white goods could potentially offer a way to better facilitate consumer engagement with issues relating to the consumption of electrical devices. However, it is first necessary to understand how such emotions are expressed and what triggers them. This was the starting point for this research. We found that people did express regret about purchasing electrical devices. Importantly, we show that ‘regret’ (and negative emotion more generally) was not only the result of poor individual choice, but fundamentally embedded in a range of everyday practices that drive socio-technical change.

Our findings also reveal that feelings of regret and negative emotion were complex, relating not only to the initial purchase of items, but also to such factors as product performance (including energy consumption, noise levels, etc.), appearance, lifespan and overall utility (table 3). On the simplest level, unhappiness with consumption was found to occur when devices did not enable or, in fact, actually inhibited the performance of desired practices. Importantly, participants also talked about the pressure to keep up with socio-technical change and discussed obsolescence, both of which necessitated the constant updating of devices (e.g. laptops, smart phones)—something that they resented *having* to do.

The fast pace of technological change in ICT equipment, for example, meant that our participants felt forced to constantly acquire new devices to enable them to benefit from the latest technological developments. In short, fast technological change was met with mixed emotions. On the one hand, participants embraced the ability to perform practices such as communicating in new (and often more convenient) ways, but still felt frustrated with what Packard [30] described as ‘functional obsolescence’. While appliances (e.g. laptops) were still *technically* operational, their performance was seen as increasingly limited as both software and expectations moved on.

As social practices evolve, the ‘equipment’ necessary to perform the practices also evolves. Cooper [35] argues that this puts significant pressure on consumers to purchase new devices, such as digital/smart televisions, or to replace or complement their existing equipment. Participants often had a wide range of devices with similar or overlapping functions (e.g. laptops, desktops, iPads and mobile phones all being used to access the internet and facilitate communication). When talking about the factors (and pressures) which had resulted in these purchases, our participants expressed regret through a number of negative emotions, such as frustration and unhappiness about reliance on a constantly evolving set of electronic devices. In essence our participants were suggesting that devices become what Packard described as *desirability obsolete* [30].

Participants also highlighted what Packard [30] described as *obsolescence of quality.* It was often either not cost effective or not technically feasible to repair items; the implication being that rather than replacing a single broken element, the whole device would need replacing. This was the case, for example, with washing machines and televisions, which participants stated they needed to replace far sooner than they had anticipated. There was a general sense that at least part of the reason why this was happening more regularly than in the past was due to manufacturers increasingly building in ‘planned obsolescence’ to modern appliances to encourage consumers to replace them more often [30].

### (b) ‘Silences’ in the data

So far, we have focused on what participants said. It is worth briefly pausing, however, to consider some of the ‘silences’ in the data—that is, the things that were not said. Interestingly, participants' comments reveal that they valued and were motivated by frugality and waste prevention. Participants did not, however, explicitly speak about valuing the environment or being motivated by environmental concern *per se*. This can also be seen in the survey, where relatively few participants selected the option which read: ‘on reflection the product did not fit with my health or environmental concerns’. Furthermore, many of the participants' positive emotions (e.g. feelings of pleasure, satisfaction) were associated with practices such as repairing a broken device, or making something last longer than they originally thought possible. In short, practising frugal behaviours was something that participants appeared to both enjoy and value (supporting the work of Kasser within this volume [36]). It is perhaps not surprising, therefore, that negative feelings were associated with wasteful behaviours and unnecessary consumption (e.g. throwing away and/or having to replace things that *should* be mendable). While it may perhaps be somewhat discouraging that our participants did not appear to be motivated specifically by environmental concerns, a number of recent publications have suggested that both frugality and voluntary simplicity may in fact be more helpful drivers of pro-environmental behaviours, and have similar end results [37–39]. Furthermore, research suggests that feelings of positive affect resulting from acting pro-environmentally (and arguably also by extension frugally) can actually motivate continued engagement with pro-environmental behaviours [40].

## 6. Policy implications

This research has demonstrated that regret and unhappiness with consumption are not simply a result of misguided individual choices. Instead, it suggests that negative emotions occur as a result of our participation in the wider socio-technical systems that constitute everyday life. As such, efforts to reduce consumption that actually make people unhappy, should not simply depend on policies geared towards persuading individuals to make sacrifices and change their behaviour [41]. Instead, policies need to also challenge the contemporary rules game and support more sustainable regimes of technologies, routines, conventions, markets, expectations and forms of knowhow [41].

In the case of the consumption of electrical devices, interventions could take a number of possible forms. Legislation governing the minimum standards of devices could include clauses requiring them to be future-proofed, thereby ensuring that they could be repaired or upgraded rather than replaced as technology changes. For example, phones could be designed to avoid obsolescence by having components which can be replaced and thereby updated. Through legislation, such designs could become the norm. Such interventions could help reduce the material burden created by the constant manufacturing of new devices [42] and also potentially challenge the dominant throwaway culture that has become entrenched in everyday practices across much of the developed world [43]. However, as Cooper notes, to make such changes it would be necessary to look more widely at the way in which markets operate and technology evolves to ensure a sustainable transition.

Legislation could also be introduced requiring manufacturers to provide consumers with details of the present and potential future benefits of upgrading to the latest version of a particular device, as well as the time scale for the release of any future upgrades. The provision of such information could help dispel the social anxiety created by manufacturers that, unless consumers obtain the latest versions of devices, their participation in everyday practices such as electronic communication will be curtailed. Furthermore, it would enable consumers to make more informed decisions about when was the optimal time to purchase a new device or upgrade an existing one without fear that it would become obsolete within a short period of time.

Finally, it is also worth noting that environmental protection should not be the only driver for such changes in legislation. This research adds to a growing body of literature which suggests that promoting environmentally sustainable practices also has significant benefits in terms of improved welfare and well-being [44–46].

In table 3, we provide a list of possible solutions to the various forms of regret (and negative emotions more generally) identified by this research. Product malfunction resulting from built-in obsolescence, for example, could be addressed by extended warranties and provision of product repair services, while disappointment at under-utilized products could be addressed by ‘try before you buy’ schemes.

## 7. Future research

Framing regretted consumption as simply being a problem of individual behaviour both restricts our understanding of the problem and excludes potential options for addressing the issues. As Elzen *et al.* [47] note, societal transformations involve not only technological artefacts but also new markets, user practices, regulations, infrastructure and cultural meanings. Consequently, future research into regretted consumption (and more general unhappiness with consumption) needs to broaden out and explore a wide range of socio-technical issues which underpin contemporary levels of consumption, in particular:

— What would the impact of improving product longevity on global markets be and how could markets be restructured to sustain fewer devices with longer lifespans?
— What are the implications of our growing reliance on an increasingly wide range of ICT equipment to perform everyday practices?
— How could feelings of resentment, regret, dissatisfaction and general unhappiness be leveraged to increase public participation in campaigns to reform industry standards?

This research focused on participants aged 35 years and older. Another avenue for future research would be to explore regret and negative emotions more generally about consumption among younger people and, perhaps, especially teenagers. Younger people are likely, for example, to feel even greater pressures to keep pace with technological change and, being at an earlier stage of life, may feel greater need to acquire goods (because they aspire to set up their own home, get a car, etc.). There is also scope to further explore questions around regret using a wider range of methodological approaches. In particular, we acknowledge Marteau's (in this volume [48]) point that many of the decisions we make about resource use are largely unconscious and to address these concerns a more innovative approach involving observation and/or social experimentation may be necessary to further develop our understanding of the negative emotions associated with unnecessary consumption.

## Data accessibility

Survey data: Yougov depository (https://d25d2506sfb94s.cloudfront.net/cumulus_uploads/document/eyx9j5gaw8/YGArchive-271016-UniversityofCam.pdf). Interview data will be publicly available by the time of publication through Dryad (doi:10.5061/dryad.s05q6).
